# The Impact of Parental Migration on Multidimensional Health of Children in Rural China: The Moderating Effect of Mobile Phone Addiction

**DOI:** 10.3390/children10010044

**Published:** 2022-12-25

**Authors:** Mi Zhou, Biyu Bian, Weiming Zhu, Li Huang

**Affiliations:** College of Economics and Management, Shenyang Agricultural University, Shenyang 110866, China

**Keywords:** parental migration, left-behind children, health, depression, cognitive ability, mobile phone addiction

## Abstract

Improving physical, mental and cognitive health is a strategic choice to help developing countries cross the middle-income trap. This paper used data from the 2019 China Rural Children Health and Nutrition Survey (*n* = 826), and used the Ordered Probit (Oprobit), Logit and ordinary least squares (OLS) analytical methods to systematically analyze the implications of parental migration on multidimensional health. The results indicate that parental migration significantly harms the physical and mental health of rural children, and that mobile phone addiction has a significant moderating effect. Moreover, parental migration has a greater impact on the physical health, mental health and cognitive ability of boys and rural children with low family income, while parents with higher nutrition knowledge and education can effectively improve the physical health and cognitive ability of their children. In conclusion, in order to improve the multidimensional health of rural children, the government should strengthen the policy of care and support for children whose parents migrate. Schools and families should pay attention to the supervision of rural children’s mobile phone addiction.

## 1. Introduction

As the Chinese economy developed after the country began to open up, more members of the agricultural labor force migrated into cities to engage in non-farm work. However, although China has promoted the development of its urban social economy, it also produces a large number of rural left-behind children [1,2] and migrant children [3]. According to the 2020 Education Statistics released by the Ministry of Education, among the students within the age range for national compulsory education (Grades 1–9), there were 12.897 million left-behind children and 14.297 million children of migrant workers in China in 2020. Parental migration negatively impacts the development of rural children, as they face problems such as malnutrition, depression and dropping out of school. Therefore, the development of their physical, mental and cognitive health poses a global challenge. The Sustainable Development Goals (SDGs) launched by the United Nations (UN) cannot be achieved without the realization of child rights, such as good health and quality education.

Many academic studies have focused on the impact of parental migration on rural physical health, mental health and cognitive ability in rural children. Most studies on left-behind children posit that parental migration is not conducive to the healthy development of rural children. Some scholars find that after their parents migrate for non-farm work, rural left-behind children are usually raised by one of their parents or cared for by their grandparents. However, intergenerational care faces many challenges. Children raised by their grandparents face problems such as unbalanced nutritional intake [4,5], loneliness [6], autism [7] or cognitive ability decline [8]. Other scholars believe that parental migration increases family income and therefore increases investment in diet, nutrition and education for these children [9,10]. Therefore, these studies argue that parental migration has no negative impact on the physical health and academic performance of children.

Research on migrant children shows that parental migration can improve their health. Compared with left-behind children, the children who migrate to cities with their parents may have better living conditions and educational environments and receive more care from their parents [11]. Therefore, these migrant children may have better physical health, mental health and cognitive ability. Through counterfactual analysis, some scholars have found that inequality of opportunity in nutritional outcomes in China would increase by more than 19% if rural children migrate to cities [12]. Migrant children are more likely to encounter junk food than left-behind children, and excessive calorie intake can cause overnutrition [13], obesity [14] or other diseases [15,16]. In addition, migrant parents have less time to help their children with their homework or accompany their children, which is not conducive to the mental health and cognitive ability of these migrant children [10,17].

The issues of parental migration, smart phone addiction, and the multidimensional health of rural children have been researched extensively in academic circles, implying that greater attention should be paid to the issues amid massive migration and urbanization in China in the years to come. There are important implications not only for individual economic and social outcomes but also for the development of human capital in China. Therefore, this paper uses representative survey data collected by the Rural Children Health and Nutrition Survey in 2019 to analyze the implications of parental migration on the multidimensional health of rural children.

### 1.1. Conceptual Framework and Hypotheses

Studies have found that parental migration causes rural children to face physical, psychological and cognitive health barriers and risk behavioral problems. Mobile phone addiction is one of these problem behaviors [18]. The migrant parents provide their children with smart phones to communicate with them, so rural children may also face a risk of mobile phone addiction [19]. According to the 47th statistical report on China’s Internet development, as of December 2020, the number of Internet users in China was 989 million, and Internet users under the age of 19 accounted for 16.6% of the total, of which children under the age of 10 accounted for 3.1%. In fact, when their parents migrate into the city for non-farm work, these children lack parental supervision and self-control, which makes them seek social support from the Internet, leading to mobile phone addiction [20].

Mobile phone addiction not only induces physiological diseases and reduces learning efficiency and academic engagement [21], but also affects interpersonal communication [22] and leads children to develop personality disorders, which seriously hinders the accumulation of human capital in rural left-behind children. Studies have shown that using mobile phones for more than seven hours a day can cause dizziness, anxiety, cognitive issues and sleep quality decline [23]. Additionally, cell phone radiation can have an inhibitory effect on brain metabolism [24]. From a psychological point of view, mobile phone addiction can exacerbate loneliness, cause psychological disorders such as anxiety and depression [25,26], and lead children to indulge in virtual worlds while ignoring real-world interpersonal communication and becoming detached from real life. When rural children face the problem of mobile phone addiction, it may aggravate the negative impact of parental migration on their health.

**Figure 1:**
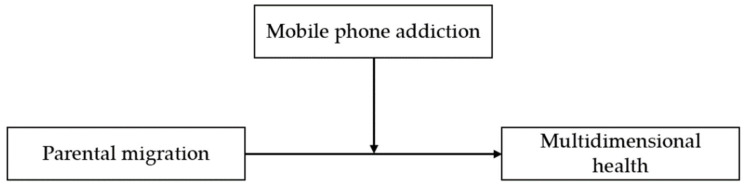
Conceptual Framework.

In summary, there is an abundance of studies on the relationship between parental migration and the health of rural children, but there are still some problems to be further discussed. Firstly, health is a state of complete physical, mental and social well-being and not merely the absence of disease or infirmity [27]. The existing literature mostly discusses health from a single perspective. This paper measures the health of rural children from multiple dimensions such as physical health, mental health and cognitive ability, which is conducive to a comprehensive evaluation of individual health, and analyzes the relationship between parental migration and the health of rural children. Secondly, this paper analyzes in depth the differences in physical health, mental health and cognitive ability between rural left-behind children and migrant children. Thirdly, this paper explores the mechanism of the impact of parental migration on the multidimensional health of rural children from the perspective of mobile phone addiction, which helps to clarify the correlation between parental migration, mobile phone addiction and the multidimensional health of rural children. The conceptual framework is shown in Figure 1.

Specifically, this paper aims to investigate the following two hypotheses:

**Hypothesis 1 (H1).**  *Parental migration has a negative impact on the multidimensional health (including physical health, mental health and cognitive ability) of rural children*.

**Hypothesis 2 (H2).**  *Mobile phone addiction regulates the impact of parental migration on the multidimensional health of rural children*.

### 1.2. Purpose

The purpose of this paper is threefold. First, this paper measures the health of rural children from multidimensional aspects including physical health, mental health and cognitive ability, which is meant to enrich the research on rural children’s human capital. Second, this study explores the impact of the migrant status (such as those children who are left behind in rural areas or those that migrate with their parents to a city) on rural children and their physical health, mental health and cognitive ability. Third, the regulatory role of mobile phone addiction on the impact of parental migration on the multidimensional health of rural children was considered.”

The remainder of this paper is organized as follows. Section 2 presents the materials and methods. Section 3 analyzes the empirical results. Section 4 discusses the implications and limitations of this paper. Section 5 draws conclusions.

## 2. Materials and Methods

### 2.1. Data

This paper uses the Rural Children Health and Nutrition Survey conducted by the Center of Agricultural Economic Theory and Policy Research of Shenyang Agricultural University in July 2019. Using the multistage cluster sampling method, these data were collected from four primary schools in three regions with different socio-economic development levels in Liaoning Province, China (i.e., Shenyang, Xinmin and Benxi City). The surveys contain standard information on the demographic characteristics of 826 rural children in grades four to six, 826 guardians and 36 head teachers, and provide information on food consumption, health, academic achievement, mobile phone addiction and household economic conditions. By eliminating the members of the sample with incomplete responses and missing key variables, the valid sample size of this paper is 826.

**Figure 2:**
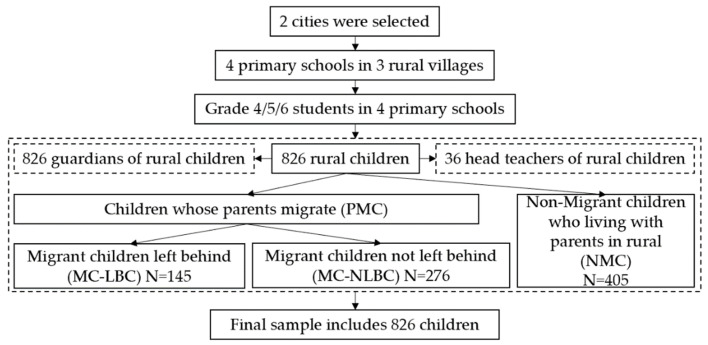
Flowchart of the sampling process.

This paper defines parental migration as when one or both parents enter the city from the countryside to engage in non-farm work for 6 or more months [19]. In the existing literature, parental migration refers to the migrant status of children, who migrate with their parents or are left behind in rural areas [28,29]. Therefore, this paper uses the word “child” to uniformly define variables from the child dimension, and defines “parental migration” as “children whose parents migrate (PMC)”. Parental migration leads to two types of rural children: migrant children left-behind (MC-LBC), who live with one or both parents in the city; and migrant children not left behind (MC-NLBC), who stay in the countryside while their parents migrate. In this paper, 51% are children whose parents migrate (PMC), 33% are migrant children not left behind (MC-NLBC) and 18% are migrant children left-behind. The flowchart of the sampling process is shown in Figure 2.

### 2.2. Variable Measures

#### 2.2.1. Multidimensional Health

In this paper, health is measured in terms of physical, mental and cognitive health.

First, physical health is mainly measured by the self-rated health of rural children, from 1 to 5 indicating “very unhealthy”, “unhealthy”, “neutral”, “healthy” and “very healthy,” respectively. As an overall health evaluation index, self-rated health status is not only highly correlated with objective indicators such as mortality and morbidity, but also contains a variety of information, such as disease severity, disease history and health status stability, which reflects the health status of individuals [30,31]. However, self-rated health is also subjective, and in order to prevent rural children from inaccurately evaluating their own health, this paper uses separate health evaluation questions for a third person with better and worse health status than the self-assessed child, and evaluates and scores the health of the third person by reading the case. Then, Anchoring Vignettes are used to revise self-rated health scores [32,33,34]. Body mass index (BMI) is another important variable to measure physical health in this paper and is the key explanatory variable in the robustness test. BMI uses the ratio between height and weight to measure whether a person is too thin or too fat, so as to judge the health of the human body [35]. Unlike self-rated health, BMI is calculated based on the actual height and weight of the respondent, which is accurate and scientific [36]. Moreover, the critical value of BMI can be used to further judge the physical health status of the interviewees. The nutritional health problems are divided into malnutrition and overnutrition. Malnutrition is characterized by growth retardation and emaciation, and overnutrition is characterized by increased weight and obesity [37].

Second, mental health, mainly measured by the Center for Epidemiologic Studies Depression (CES-D), is evaluated. The CES-D scale has been widely used in related research to assess depressed mood and depression [38]. The scale consists of 20 questions assessing four aspects of individual depression and is scored on a Likert scale. The CES-D scale asks respondents to self-assess how often a certain mood has occurred in the past week, with 0 to 3 indicating “hardly ever (less than one day)”, “Some of the time (1–2 days)”, “Often (3–4 days)” and “Most of the time (5–7 days)”, respectively. Of these, 16 questions asked about the frequency of negative emotions, and 4 asked about the frequency of positive emotions (questions 4, 8, 12 and 16). The scores of the four positive questions were reversed and the sum of their scores was used as the depression score variable. Possible scores range from 0 to 60 and a score of 17 or higher is indicative of depression [39]. In addition to determining whether or not children exhibit depressive symptoms, this paper uses the CES-D score to determine the severity of depression in rural children. Specifically, this paper sets three levels of depression severity: a CES-D score between 17 and 23 indicates “mild depression”, a score between 24 and 28 indicates “moderate depression” and scores of 29 or more indicate “severe depression.” Depression has been extensively measured using the CES-D scale, and the scale has passed the reliability and validity tests for the Chinese population [40]. Therefore, the scale is applicable to the measurement of the mental health of children in rural China.

Third, academic achievement is used as a proxy variable for cognitive ability in this paper. Cognitive ability refers to intelligence and problem-solving skills, including understanding, memory, reasoning and thinking. It is generally measured with IQ tests, academic performance and other indicators [41]. Cognitive ability includes both the traditional curriculum skills of language and mathematics, as well as logical thinking and imagination [42]. In existing studies, academic achievement is often used to measure the cognitive ability of rural children [43]. However, due to the inconsistency of question-setting standards and test difficulty, academic achievement may not be comparable. However, the number of students in all grades in the school is relatively stable [44]. Therefore, academic rankings more accurately reflect the cognitive ability of rural children. In this paper, cognitive ability is measured using their rankings in their Chinese, mathematics and English classes.

In order to make the academic achievement of rural children of different grades in the sample comparable, this paper ranks them from high to low in the same school, the same grade, and the same class according to their scores in Chinese mathematics, and English classes. The average of these scores is considered, but was not significant for this sample. This paper divides the ranking into five groups: top 20% are excellent (1 = excellent, 0 = other), top middle 20% are very good (1 = very good, 0 = other), middle 20% are medium (1 = medium, 0 = other), lower middle 20% are poor (1 = poor, 0 = other) and bottom 20% are very poor (1 = very poor, 0 = other) [45]. In addition, the ranking can be compared on the premise that the classes are randomly assigned; that is, there is no difference between good classes and poor classes, and the quality of teachers is similar. Therefore, we also set up a random assignment test to check the accuracy of the academic performance ranking. The results show that the coefficients of most variables are not significant, and there is no positive significant correlation, whether based on the characteristics of the students (Table A2) or the teachers (Table A2), indicating that students and teachers are randomly assigned to each class [46,47]. Therefore, academic performance ranking used in this paper is accurate.

#### 2.2.2. Mobile Phone Addiction

Mobile phone addiction was assessed using the Smart-phone Addiction Scale (SAS), a modified 22-item scale related to the study of adolescent cyber psychology and behavior [48]. The SAS scale includes six aspects: individual withdrawal behavior, emergent behavior, social appeasement, negative influence, app use and app updates [49]. Among them, withdrawal behavior refers to negative psychological or behavioral reactions when not engaging in mobile phone activities; emergent behavior refers to smartphone use occupying the center of thinking and behavioral activities; social appeasement refers to the role of smartphone use in interpersonal interactions; negative effects refer to decreased work and study productivity due to excessive smartphone use; app use refers to excessive use of smartphone applications; and app updates refer to the excessive attention of smartphone users to app updates. The scale was scored using a five-point Likert scale, with 1–5 indicating “very inconsistent”, “inconsistent”, “uncertain”, “consistent” and “very consistent”, respectively [48]. The scores for all 22 items were added up (for a range of 22 to 110). A total score of 66 or higher indicates the presence of mobile phone addiction. The reliability and validity of the SAS scale have been established in Chinese children, and the Cronbach’s alpha was 0.904 and the KMO was 0.933 in the present study.

### 2.3. Statistical Analysis and Model Specification

#### 2.3.1. Statistical Analysis

This paper presented socio-demographic characteristics of rural children and their guardians in mean and standard deviation. T-tests were employed to examine multidimensional health, including physical health, mental health and cognitive ability among children whose parents migrate (PMC), migrant children left-behind (MC-LBC), migrant children not left behind (MC-NLBC) and non-migrant children who live with their parents in rural areas (NMC). The mean difference was tested to determine if physical health, mental health and cognitive ability differ statistically (with *p* < 0.05) among PMC, MC-LBC, MC-NLBC and NMC.

#### 2.3.2. Model Specification

Referring to the existing research, the dependent variable of this paper, the health of rural children, is analyzed from three main perspectives: physical health, mental health and cognitive ability. An Ordered Probit model was adopted to identify the impact of parental migration on the physical health, mental health and cognitive ability of rural children, because the dependent variables were ordered data. Anchored self-rated health and BMI are very important indicators for measuring the physical health of children. The estimation strategies for the impact of parental migration on the physical health of rural children are as follows:

(1) $$physicalhealth_{i}=\alpha +\beta_{1}migrant+\beta_{2}cmigtype+\beta_{3}addiction+\beta_{4}migrant\times addiction+X_{i}\Gamma +p_{j}+c_{k}+g_{i}+\varepsilon_{i}$$

where *i* represents the *i*-th child, $physicalhealth_{i}\;$ is physical health, migrant indicates the dummy variable of parental migration, cmigtype indicates the dummy variable of child type (1 = MC-NLBC, 0 = MC-LBC); addiction is the degree of addiction to mobile devices; migrant×addiction represents the interaction between parental migration and mobile phone addiction; $X_{i}$ represents the control variables, including individual characteristics (age of children, brothers and sisters, boarding, nutritious lunch, age of parent, education of parent, personality of parent and nutritional cognition) and family characteristics (distance from home to school, annual family income). $\beta_{1}$, $\beta_{2}$, $\beta_{3}$ and $\beta_{4}$ are the vectors of the estimated parameter; $p_{j}$, $c_{k}$ and $g_{i}$ indicate the dummy variables of region, school and grade.

CES-D depression score and depression degree are widely used to measure mental health. The estimation strategies for the impact of parental migration on the mental health of rural children are as follows:

(2) $$mentalhealth_{i}=\alpha +\beta_{1}migrant+\beta_{2}cmigtype+\beta_{3}addiction+\beta_{4}migrant\times addiction+X_{i}\Gamma +p_{j}+c_{k}+g_{i}+\varepsilon_{i}$$

In the above formula, $mentalhealth_{i}$ is the mental health of rural children, migrant indicates the dummy variable of parental migration, cmigtype indicates the dummy variable of child type (1 = MC-NLBC, 0 = MC-LBC); addiction is the degree of addiction to mobile devices; migrant×addiction represents the interaction between parental migration and mobile phone addiction; $X_{i}$ represents the control variables, including individual characteristics (age of children, brothers and sisters, boarding, nutritious lunch, age of parent, education of parent, personality of parent and nutritional cognition) and family characteristics (distance from home to school, annual family income). $\beta_{1}$, $\beta_{2}$, $\beta_{3}$ and $\beta_{4}$ are the vectors of the estimated parameter; $p_{j}$, $c_{k}$ and $g_{i}$ indicate the dummy variables of region, school and grade.

The estimation strategies for the impact of parental migration on the cognitive ability of rural children are as follows:

(3) $$cognitive_{i}=\alpha +\beta_{1}migrant+\beta_{2}cmigtype+\beta_{3}addiction+\beta_{4}migrant\times addiction+X_{i}\Gamma +p_{j}+c_{k}+g_{i}+\varepsilon_{i}$$

In the above formula, $cognitive_{i}$ represents the mental health of rural children, migrant is the dummy variable of parental migration, cmigtype indicates the dummy variable of child type (1 = MC-NLBC, 0 = MC-LBC); addiction is the degree of addiction to mobile devices; migrant×addiction represents the interaction between parental migration and mobile phone addiction; $X_{i}$ represents the control variables, including individual characteristics (age of children, brothers and sisters, boarding, nutritious lunch, age of parent, education of parent, personality of parent and nutritional cognition) and family characteristics (distance from home to school, annual family income). $\beta_{1}$, $\beta_{2}$, $\beta_{3}$ and $\beta_{4}$ are the vectors of the estimated parameter; $p_{j}$, $c_{k}$ and $g_{i}$ indicate the dummy variables of region, school and grade.

## 3. Results

### 3.1. Descriptive Statistics Results

A total of 826 rural children and 826 parents and guardians were used in this sample. The proportion of children whose parents migrate was 51%, with migrant children left-behind and migrant children not left-behind making up 33% and 18%, respectively. In terms of individual characteristics, boys and girls were equally represented among rural children, and their average age was 11.70. Their average mobile phone addiction score was 48.82. In addition, 46% of rural children have brothers and sisters, 2% of them board at school, and 81% of them eat a nutritious lunch at school. The average age of the parents and guardians was 40.85, they had an average of 9.14 years of education, and their average nutrition knowledge score was 50.40. In terms of parent personality, the mean values for conscientiousness, agreeableness, emotional stability, extroversion and openness were 11.28, 11.10, 7.70, 9.87 and 9.73, respectively. The average distance from home to school was 2.43 km, and the average annual family income was 45,700 yuan.

It can be seen from Table 1 that the multidimensional health of rural children is good. Specifically, in terms of physical health, the average level of self-rated health modified by Anchoring Vignettes was 3.72 with a range from 1 to 5. Furthermore, BMI was calculated according to the height and weight of rural children in order to objectively evaluate their health status. In this paper, 33.66% of rural children had a normal BMI, but 49.76% of rural children were thin, and 16.59% of rural children were overweight or obese. In terms of mental health, the average CES-D depression score of rural children is 16.82, which is less than the critical value of depression (17), indicating that most rural children had good mental health. However, 18.28% of rural children had mild depression, 11.14% had moderate depression, and 13.08% had severe depression. From the perspective of cognitive ability, the average ranking of academic achievement has five levels, the proportion of excellent, very good, medium, poor and very poor were 18.8%, 20.3%, 19.7%, 20.3% and 20.8%, respectively.

This paper further analyzes multidimensional health differences in children whose parents migrate to cities for non-farm work, children living with parents in the countryside, migrant children and left-behind children using a *t*-test. The results of the *t*-test are shown in Table 2.

Generally speaking, the physical health (anchored self-rated health diff = −0.25, *p* < 0.05) and mental health (CES-D score diff = 2.91, *p* < 0.05; the Depression level diff = 0.23, *p* < 0.05) of migrant children left-behind (MC-LBC) is the worst, but their cognitive ability is the best (Table 2 columns 4–5). Migrant children not left-behind (MC-NLBC) have the best physical and mental health, but the worst cognitive health (Table 2 columns 6–7). A possible reason is that migrant children not left-behind (MC-NLBC) not only enjoy the good living conditions brought about by an increase in family income, but also receive better care from their parents, so their physical and mental health is the best. However, the migrant children not left-behind (MC-NLBC) transferred from rural schools to urban schools, and the change in teaching methods and curriculum led to a decline in their grades.

### 3.2. Baseline Regression Results

#### 3.2.1. The Impact of Parental Migration on the Multidimensional Health of Rural Children

To investigate how parental migration affects the multidimensional health of rural children, the values of physical health, mental health and cognitive ability were estimated. Table 3 shows the results of the estimation using the Oprobit model.

Our results show that parental migration has a significant negative impact on the physical health of rural children. The coefficients are −0.24 and −1.08, and the *p* values are less than 0.01 (Table 3 columns 1–2). This finding indicates that the anchored self-rated health of rural children will decline when parents migrate. However, further considering the migrant children who migrate with their parents, this paper finds that the anchored self-rated health of migrant children is better than left-behind children, with a coefficient of 0.20 (*p* < 0.01).

Parental migration negatively affects the mental health of rural children. The dependent variable is the depression level of rural children: 0–3 indicates “no depression” to “severe depression.” The higher the value, the higher the level of depression. According to the results (Table 3 columns 3–4), parental migration significantly increases the depression level of rural children. The coefficients are 0.22 and 1.38 (*p* < 0.01). However, while children migrating with their parents may improve their depression level, the difference is not statistically significant.

Parental migration has a significant positive effect on improving the cognitive ability of rural children. Columns 5–6 of Table 3 report the regression results. The table indicates that parental migration significantly improves the mathematics ranking and English ranking of rural children, as the coefficients are 2.20 and 1.11 (*p* < 0.01). However, there is no significant impact on their Chinese ranking. In addition, migrant children have lower Chinese, mathematics and English rankings, with coefficients of −0.25, −0.14 and −0.04 (*p* < 0.01), respectively.

#### 3.2.2. The Moderating Effect of Mobile Phone Addiction

This paper further explores the moderating effect that mobile phone addiction plays in the impact of parental migration on the multidimensional health of rural children. Columns 2, 4 and 5–7 of Table 3 show the regression results of the interaction between parental migration and mobile phone addiction. It can be seen from the results that, compared with column 1 and column 2 of Table 3, the absolute value of the regression coefficient of parental migration on the anchored self-rated health, depression level and academic achievement ranking of rural children increases, showing that when parental migration and mobile phone addiction of rural children occur at the same time, parental migration has a more significant impact on the multidimensional health of rural children. Therefore, mobile phone addiction plays a significant moderating role in the impact of parental migration on the health of these children. It is worth noting that mobile phone addiction has a significant negative impact on health, so parents should improve their awareness of the harm of mobile phone addiction and make sure to supervise their children.

The interaction between parental migration and mobile phone addiction has a significant positive impact on the physical and mental health of rural children, but has a significant negative impact on their cognitive ability. The moderating effect of mobile phone addiction includes the following three aspects. Firstly, the regression coefficient of the interaction between parental migration and mobile phone addiction on the anchored self-rated health of rural children was 0.22 (*p* < 0.01). Secondly, the regression coefficient of the interaction between parental migration and mobile phone addiction on the depression level of rural children was −0.30 (*p* < 0.01). This shows that mobile phone addiction can significantly reduce the depression level of rural children when their parents migrate. Thirdly, the regression coefficient of the interaction between parental migration and mobile phone addiction on the cognitive ability of rural children is negative, but it only has a significant impact on mathematics and English rankings.

### 3.3. Robustness Check

In order to test the robustness of the results, this paper uses BMI, CES-D depression score and total ranking of academic achievement as dependent variables to measure the physical health, mental health and cognitive ability of rural children. Logit, OLS and Oprobit models were used to analyze the impact of parental migration on the multidimensional health of rural children, and to explore the moderating effect of mobile phone addiction. The regression results of the robustness test are shown in Table 4. After replacing the dependent variables and the estimation model, parental migration still has a significant impact on the multidimensional health of rural children, indicating that the results of this paper are robust.

It can be seen from Table 4 that after the replacement of explained variables, parental migration still has a significant impact on the multidimensional health of rural children, indicating that the results of this paper are relatively robust. In terms of physical health, parental migration increases the probability of abnormal BMI, as the coefficient is −2.45 (*p* < 0.01). In terms of mental health, the CES-D depression score of rural children increased by 0.23 units (*p* < 0.01) when their parents migrated for non-farm work. In terms of cognitive ability, parental migration significantly improves total academic achievement, with a regression coefficient of 1.41 (*p* < 0.01), indicating that parental migration contributes to improving the cognitive ability of rural children. Migrating with parents into cities improves the physical and mental health of rural children to a certain extent, but also reduces their cognitive ability, which is consistent with the previous regression results. At the same time, the moderating effect of mobile phone addiction still holds. Mobile phone addiction significantly reduces the physical health, mental health and cognitive ability of rural children. The interaction between parental migration and mobile phone addiction is beneficial to improving the physical and mental health of rural children, but significantly reduces their cognitive ability.

### 3.4. Heterogeneity Analysis

Because of the widespread preference for sons in rural China, girls face gender inequality in health care, access to food and education. Therefore, this paper regressed the samples of boys and girls, respectively, to explore the possible gender heterogeneity of the impact of parental migration work on the multidimensional health of rural children. As can be seen from Columns 1–2 of Table 5, parental migration has a significant negative impact on the physical health of both boys and girls, but boys are more likely to have abnormal BMI than girls. In terms of mental health, migrant work significantly increased depression in boys, but not in girls. In terms of cognitive ability, having parents who work outside the home raises the overall academic ranking of boys and girls.

Considering the different situations of children in families with different family incomes [50], this study divided the sample into high-income and low-income families according to family income to explore the heterogeneity of the multidimensional health effects. As shown in Table 5, parental migration has a significant negative impact on the physical health of rural children from both high-income and low-income families, but children from low-income families are more likely to face abnormal BMI. Poor access to diverse foods and balanced nutrition in low-income households, as well as lack of care for migrant parents, may contribute to poorer physical health among rural children. From the perspective of mental health, the degree of depression of children from low-income families is significantly increased by their parental migration, but it has no significant effect on high-income families. From the perspective of cognitive ability, parental migration significantly improves the overall academic ranking of children from high-income families but has no significant impact on children from low-income families. The higher the income, the more the family will invest in education, which is conducive to improving the academic performance of rural children.

Many studies show that the health of rural children is transmitted between generations [51,52,53]. Therefore, this paper further investigates the differences in nutritional cognition, personality characteristics and educational level of guardians, and analyzes the differences in the impact of parental migration on physical health, mental health and cognitive ability. As can be seen in Table 5, for children who lack nutritional knowledge, having their parents be migrant workers significantly increases their risk of abnormal BMI. Both the group whose guardians lack nutritional knowledge and the group whose guardians have adequate nutritional knowledge, experience improved cognitive ability through parental migration.

As can be seen in Table 5, if the guardian has a low level of education, having their parents become migrant workers will significantly increase their risk of abnormal BMI, which is not conducive to their health. From the perspective of mental health, for the group with high guardian education level, parental migration significantly reduces the depression scores of rural children, which is conducive to improving their mental health. However, for the group with low guardian educational attainment, parental migration will significantly increase their depression scores, which is not conducive to their mental health. From the perspective of cognitive ability, if the children have guardians with high educational attainment, parental migration will significantly improve their overall academic ranking and thus improve their cognitive ability, but parental migration has no significant impact on the cognitive ability of rural children if their guardians have a low level of education.

## 4. Discussion

This paper is innovative in research content and research perspective. This paper is based on a novel perspective of mobile phone addiction, and deeply analyzes the relationship between parental migration and the multidimensional health of rural children.

There is a significant correlation between parental migration and the multidimensional health of rural children. First, there is a significant negative impact on the physical health of rural children when their parents migrate for non-farm work. Specifically, the anchored self-rated health of rural children decreased by 1.08 units, which is consistent with existing research conclusions [54,55]. Compared with left-behind children, the physical health of migrant children is better. One possible reason is that when children migrate with their parents, the higher family income effectively promotes their access to better nutrition and medical services [56]. At the same time, parental care is more conducive to them developing healthy eating and living habits [11]. Therefore, migration with parents will significantly promote the physical health of rural children.

Secondly, parental migration has a significant negative impact on the mental health of rural children. Parental migration increases depression risk among rural children, which is consistent with the existing research conclusions [57]. A possible reason is that rural children may live with their grandparents in the countryside after their parents migrate to the city, and may lack care from their parents [58].

Finally, parental migration has a significant positive impact on improving cognitive ability. In particular, it helps to improve the mathematics and English scores of rural children. One possible reason is that family income increases greatly when parents migrate for non-farm work, so parents invest more in education for their children, such as by providing tutoring, stationery and learning materials [10]. In addition, the Chinese, mathematics and English scores of migrant children are worse than those of left-behind children. This may be due to the differences in teaching methods and content between urban and rural schools [59].

Mobile phone addiction plays a significant moderating role in the impact of parental migration on the multidimensional health of rural children. While mobile phones provide an avenue for convenient emotional communication between migrant parents and children, they can also lead to potential mobile phone addiction problems for rural children [19]. Long-term overuse of mobile phones may occupy time that would otherwise be used for exercise, study, sleep and face-to-face social interaction with peers, resulting in decreased physical health and academic performance, anxiety or depression [60]. The results of this study show that there is a substitution relationship between mobile phone addiction and parental migration. Mobile phone addiction weakens the negative impact of parental migration on the physical and mental health of rural children, but increases the negative impact on their cognitive ability. Parents may supervise their children more carefully and reduce the time they spend using mobile phones, while encouraging them to increase their exercise time [61]. However, exercise apps on mobile phones can be used to promote physical exercise among rural children. Relevant research shows that most college students use exercise apps every week, and athletics programs can improve motivation and desire to exercise [62]. Additionally, migrant parents provide their children with mobile phones to communicate with them, while left-behind children meet their inner needs and obtain satisfaction using mobile phones [63]. As left-behind children spend less time with their parents, they will be able to seek support and help from their peers. Mobile phones are used for communication and can be used in a group, which increases their utility [64].

This paper also has some limitations. The model used only analyzes the correlation between variables, so it is impossible to know the exact causal relationship between parental migration and the multidimensional health of rural children. A more in-depth theoretical study and empirical analysis are necessary. In addition, some factors that contribute to the decision of parental migration are not observable in this paper. For example, parents who believe that the health of their children would suffer may have decided not to migrate. Therefore, there may be selection bias. The possible endogenous problems in this paper deserve further discussion.

## 5. Conclusions

This paper uses the 2019 China Rural Children Health and Nutrition Survey (CRCHNS) data to analyze the implications of parental migration on the multidimensional health of rural children. Compared with children who live with their parents in rural China, children whose parents migrate to the city for non-farm work have worse physical health, mental health and cognitive ability. Among all rural children, 86.20% have good self-rated health, but 8.6% and 7.99% of them have a BMI that is considered overweight or obese, respectively. In terms of mental health, 42.50% of rural children are depressed, and about 15% of them have mobile phone addiction. The baseline regression and robustness check regression results indicate that (1) Parental migration significantly impacted the physical and mental health of rural children, but significantly improved their cognitive ability. (2) There is a moderating effect of mobile phone addiction, specifically, the interaction between parental migration and mobile phone addiction is conducive to improving the physical and mental health of rural children, but significantly reduces their cognitive ability. (3) Children migrating with their parents have improved physical health, but weaker cognitive ability. (4) Heterogeneity analysis shows that the impact of parental migration on the multidimensional health of rural children varies with gender, family income level, and guardian nutritional cognition, personality and education.

## Figures and Tables

**Table 1:** Sociodemographic characteristics of the sample (*n* = 826).

Dependent Variables	Variable Definition	Mean	SD	Min	Max
**Physical Health**					
Self-rated health	1–5 from “very unhealthy” to “very healthy”	3.72	1.18	1	5
BMI ^a^	1 = thin, 2 = normal, 3 = overweight, 4 = obese	1.75	0.92	1	4
**Mental Health**					
CES-D score	The higher the score, the more severe of depressive symptoms	16.82	10.24	0	57
Depression severity					
no depression	1 = Yes, 0 = No	0.575	0.495	0	1
mild depression	1 = Yes, 0 = No	0.183	0.387	0	1
Moderate depression	1 = Yes, 0 = No	0.111	0.315	0	1
severe depression	1 = Yes, 0 = No	0.131	0.337	0	1
**Cognitive Ability**					
Ranking of academic achievement					
Excellent	1= Yes, 0 = No	0.188	0.391	0	1
Very good	1 = Yes, 0 = No	0.203	0.403	0	1
Medium	1 = Yes, 0 = No	0.197	0.398	0	1
Poor	1 = Yes, 0 = No	0.203	0.403	0	1
Very poor	1= Yes, 0 = No	0.208	0.406	0	1
**Independent Variables**					
Children whose parents migrate	1 = Yes, 0 = No	0.51	0.50	0	1
Migrant children left-behind	1 = Yes, 0 = No	0.18	0.38	0	1
Migrant children not left-behind	1 = Yes, 0 = No	0.33	0.47	0	1
**Moderator Variables**					
Mobile phone addiction	The higher the score, the more severe mobile phone addiction	48.82	15.91	22	110
**Control Variables**					
Gender of child	1 = male, 0 = female	0.50	0.50	0	1
Age of child	Years	11.70	1.01	9	15
Siblings	1 = Yes, 0 = No	0.46	0.50	0	1
Boarding	1 = Yes, 0 = No	0.02	0.14	0	1
Nutritious lunch	1 = Yes, 0 = No	0.81	0.39	0	1
Age of guardian	Years	40.85	7.62	30	81
Guardian educational attainment	Years	9.14	2.52	0	22
Personality of guardians					
Conscientiousness	Measured by the Big Five Personality Scale	11.28	2.52	3	15
Agreeableness	Measured by the Big Five Personality Scale	11.10	2.57	3	15
Emotional stability	Measured by the Big Five Personality Scale	7.70	2.54	3	15
Extroversion	Measured by the Big Five Personality Scale	9.87	2.61	3	15
Openness	Measured by the Big Five Personality Scale	9.73	3.16	3	15
Nutritional knowledge	The higher the score, the greater the knowledge of nutritional intakes	50.40	6.36	32	68
Distance from home to school	km	2.43	3.61	0.1	30
Annual family income	10,000 yuan	4.57	3.76	1	20

Note: Data are from the China Rural Children Health and Nutrition Survey (CRCHNS) conducted in July 2019 in China. ^a^ BMI = Weight(kg)/height2 (m), where BMI ≤ 18.5 is thin, 18.5< BMI ≤ 23.9 is normal, 23.9 < BMI <= 27.9 is overweight, and BMI >= 27.9 is obese.

**Table 2:** Differences in multidimensional health between different categories of children.

Variables	NMC ^a^	PMC ^b^	Diff ^e^	MC-LBC ^c^	Diff ^e^	MC-NLBC ^d^	Diff ^e^
	*n* = 405	*n* = 421		*n* = 145		*n* = 276	
	(1)	(2)	(3) = (2) − (1)	(4)	(5) = (3) − (1)	(6)	(7) = (6) − (1)
Anchored self-rated health	3.8	3.63	−0.17 **	3.55	−0.25 **	3.67	−0.13
CES-D score	15.96	17.64	1.68 **	18.87	2.91 ***	16.99	1.03
Depression severity	0.72	0.87	0.16 **	0.94	0.23 **	0.84	0.12
Chinese ranking	2.91	3.17	0.26 ***	3.04	0.14	3.24	0.33 ***
Mathematics ranking	2.94	3.14	0.20 ***	3.19	0.25 *	3.11	0.17
English ranking	2.96	3.12	0.16	3.21	0.25 *	3.07	0.11

Note: Data are from the China Rural Children Health and Nutrition Survey (CRCHNS) conducted in China in July 2019; ^a^ NMC indicates non-migrant children who live with their parents in rural areas; ^b^ PMC refers to children whose parents migrate; ^c^ MC-LBC indicates migrant children left-behind in rural areas; ^d^ MC-NLBC refers to migrant children not left-behind; ^e^ Diff indicates the mean differences in multidimensional health between different categories of children and non-migrant children who live with their parents in rural areas (NMC); *** *p* < 0.01, ** *p* < 0.05, * *p* < 0.10. Compared with non-migrant children who live with their parents in rural areas (NMC), the children whose parents migrated (PMC) to the city for non-farm work had lower physical, mental and cognitive health (Table 2 columns 1–3). In particular, the mean difference between anchored self-rated health was 0.17 (*p* < 0.05), and the mean differences between CES-D depression score and depression level were 1.68 (*p* < 0.05) and 0.16 (*p* < 0.05), respectively. Compared with NMC, PMC have higher academic achievement rankings in Chinese and Mathematics, but have no significant difference in English.

**Table 3:** Results of parental migration impact on the health of rural children.

Variables	Physical Health		Mental Health		Cognitive Ability		
	Anchored Self-Rated Health		Depression Level		Chinese Ranking	Mathematics Ranking	English Ranking
	(1)	(2)	(3)	(4)	(5)	(6)	(7)
Children whose parents migrate	−0.24 ***	−1.08 ***	0.22 ***	1.38 ***	1.00	2.20 ***	1.11 ***
	(0.02)	(0.24)	(0.02)	(0.21)	(0.69)	(0.60)	(0.35)
Migrant children not left-behind	0.20 ***	0.20 ***	−0.04	−0.04	−0.25 ***	−0.14 ***	−0.04 ***
	(0.02)	(0.02)	(0.05)	(0.05)	(0.02)	(0.03)	(0.01)
Mobile phone addiction	−0.17 ***	−0.28 ***	1.00 ***	1.16 ***	−0.45 ***	−0.30 ***	−0.41 ***
	(0.06)	(0.01)	(0.11)	(0.05)	(0.03)	(0.04)	(0.02)
Parental migration##Mobile phone addiction		0.22 ***		−0.30 ***	−0.28	−0.61 ***	−0.33 ***
		(0.06)		(0.05)	(0.18)	(0.15)	(0.08)
Control variables	Yes	Yes	Yes	Yes	Yes	Yes	Yes
Region dummies	Yes	Yes	Yes	Yes	Yes	Yes	Yes
School dummies	Yes	Yes	Yes	Yes	Yes	Yes	Yes
Grade dummies	Yes	Yes	Yes	Yes	Yes	Yes	Yes
Observations	826	826	826	826	826	826	826

Note: Data from the China Rural Children Health and Nutrition Survey (CRCHNS) conducted in China in July 2019; *** *p* < 0.01; Cluster robust standard errors in parentheses; Control variables include age of children, number of siblings, boarding, nutritious lunch, age of parent, education of parent, personality of parent (e.g., conscientiousness, agreeableness, emotional stability, extroversion and openness), nutritional cognition, distance from home to school, and annual family income.

**Table 4:** Robustness check results.

Variables	BMI Abnormal	CES-D Score	Average Academic Ranking
	Logit	OLS	Oprobit
	(1)	(2)	(3)
Children whose parents migrate	−2.45 **	0.23 ***	1.41 ***
	(0.99)	(0.02)	(0.48)
Migrant children not left-behind	0.13 ***	−0.12 **	−0.23 ***
	(0.01)	(0.02)	(0.01)
Mobile phone addiction	−0.31 ***	0.63 ***	−0.49 ***
	(0.10)	(0.03)	(0.01)
Parental migration##Mobile phone addiction	0.59 **	−0.01	−0.39 ***
	(0.26)	(0.01)	(0.12)
Control variables	Yes	Yes	Yes
Region dummies	Yes	Yes	Yes
School dummies	Yes	Yes	Yes
Grade dummies	Yes	Yes	Yes
Constant	0.09	0.86	
	(1.13)	(0.44)	
R-squared		0.17	
Observations	826	826	826

Note: Data are from the China Rural Children Health and Nutrition Survey (CRCHNS) conducted in China in July 2019; *** *p* < 0.01, ** *p* < 0.05; Cluster robust standard errors in parentheses; Control variables include age of children, number of siblings, boarding, nutritious lunch, age of parent, education of parent, personality of parent (e.g., conscientiousness, agreeableness, emotional stability, extroversion and openness), nutritional cognition, distance from home to school, annual family income.

**Table 5:** Heterogeneity analysis results.

Variables	BMI Abnormal		CES-D Score		Average Academic Ranking	
	*Panel A: Child Gender*					
	Boys	Girls	Boys	Girls	Boys	Girls
	(1)	(2)	(3)	(4)	(5)	(6)
Children whose parents migrate	−2.21 *	−1.96 **	0.23 ***	0.15 **	1.12 ***	1.60 ***
	(1.21)	(0.99)	(0.02)	(0.02)	(0.17)	(0.56)
Constant	−0.06	1.85	1.22 ***	0.52		
	(0.72)	(1.82)	(0.11)	(1.02)		
Observations	414	412	414	412	414	412
R-squared			0.16	0.23		
**Panel B: Family Income**						
	High	Low	High	Low	High	Low
Children whose parents migrate	−1.85 ***	−3.85 *	−0.48	0.57 **	1.61 ***	1.47
	(0.56)	(2.25)	(0.25)	(0.07)	(0.17)	(1.02)
Constant	1.34	0.25	0.23	1.26		
	(1.57)	(1.71)	(0.55)	(0.59)		
Observations	349	477	349	477	349	477
R-squared			0.25	0.14		
**Panel C: Nutrition Knowledge of Guardian**						
	High	Low	High	Low	High	Low
Children whose parents migrate	−1.56 **	−3.54 ***	0.44	−0.05	1.24 ***	1.15 ***
	(0.78)	(0.74)	(0.32)	(0.18)	(0.40)	(0.34)
Constant	1.87	1.49	0.43	1.02		
	(2.15)	(1.42)	(0.61)	(0.45)		
Observations			0.19	0.17		
R-squared	431	395	431	395	431	395
**Panel D: Education of Guardian**						
	High	Low	High	Low	High	Low
Children whose parents migrate	−1.56	−2.72 **	−1.02 **	0.61 **	4.96 ***	0.37
	(1.47)	(1.23)	(0.23)	(0.09)	(1.23)	(1.02)
Constant	−3.48	2.05	1.67	0.48		
	(4.50)	(2.24)	(0.70)	(0.33)		
Observations	185	639	185	639	185	639
R-squared			0.29	0.16		
Control variables	Yes	Yes	Yes	Yes	Yes	Yes
Region dummies	Yes	Yes	Yes	Yes	Yes	Yes
School dummies	Yes	Yes	Yes	Yes	Yes	Yes
Grade dummies	Yes	Yes	Yes	Yes	Yes	Yes

Note: Data are from the China Rural Children Health and Nutrition Survey (CRCHNS) conducted in China in July 2019; *** *p* < 0.01, ** *p* < 0.05, * *p* < 0.10; Cluster robust standard errors in parentheses; Control variables include age of children, number of siblings, boarding, nutritious lunch, age of parent, education of parent, personality of parent (e.g., conscientiousness, agreeableness, emotional stability, extroversion and openness), nutritional knowledge, distance from home to school, and annual family income.

## Data Availability

Data are available from the authors upon reasonable request.

## Appendix A

In order to verify the reliability of the “random class assignment” of the sample, we tested it from the characteristics of students and teachers, respectively. Table A1 examines the correlation between the individual prerequisite variables and the characteristics of other students in the class from the full sample and the graded sample. If the assumption of random class assignment within the school is established, after controlling the fixed effect of school grade, the individual’s antecedent characteristic variable has nothing to do with the peer’s average characteristic; that is, the individual’s antecedent characteristic variable regresses the peer’s mean value of this variable, and its regression coefficient should not be significant. Table A1 lists the coefficients for regression of the mean value of the corresponding antecedent characteristic variables of the peer with each background characteristic of the individual as the dependent variable. On the whole, there is no significant positive selection between students and their peers.

**Table A1:** Random assignment test of Children.

Outcomes	Full Sample	Grade 4	Grade 5	Grade 6
	(1)	(2)	(3)	(4)
Gender of child	−0.009	−0.026 **	−0.020 *	−0.016 **
	(0.005)	(0.006)	(0.007)	(0.002)
Age of child	0.037 **	−0.014	0.029	−0.007
	(0.018)	(0.010)	(0.031)	(0.010)
Siblings	−0.006	0.002	−0.008	−0.018 ***
	(0.006)	(0.026)	(0.005)	(0.001)
Boarding	0.000	−0.019 *	0.005	−0.006
	(0.006)	(0.006)	(0.008)	(0.015)
Left-behind Children	−0.001	−0.013	−0.001	−0.015 *
	(0.007)	(0.016)	(0.012)	(0.004)
Age of guardian	0.003	−0.029 ***	−0.004	−0.005
	(0.009)	(0.002)	(0.013)	(0.007)
Education of guardian	0.026 *	−0.010	−0.022 **	0.026 *
	(0.014)	(0.014)	(0.004)	(0.007)
Annual family income	0.018 *	−0.011	−0.008	−0.020 **
	(0.010)	(0.010)	(0.008)	(0.004)
*N*	826	826	826	826
School FEs	Yes	Yes	Yes	Yes
Class FEs	Yes	Yes	Yes	Yes

Note: Data from China Rural Children Health and Nutrition Survey (CRCHNS) in China on July 2019; *** *p* < 0.01, ** *p* < 0.05, * *p* < 0.10.

In order to test whether teachers are randomly assigned to each class, Table A2 reports the regression coefficients of the mean of each background feature of the class students and their parents by each prerequisite feature (head teacher’s gender, head teacher’s age and head teacher’s teaching age) of the head teacher after controlling the fixed effect of the school, grade and class. The results show that the regression coefficients are not significant, indicating that there is no significant correlation between the characteristics of teachers and the characteristics of students in the class and their parents in the school sample randomly divided into classes.

**Table A2:** Random assignment test of Head Teacher.

**Outcomes**	**Gender of Head Teacher**	**Age of Head Teacher**	**Teaching Age of Head Teacher**
	**(1)**	**(2)**	**(3)**
Gender of child	0.733	−20.836	−17.951
	(0.867)	(15.030)	(16.048)
Age of child	0.018	−1.316	−0.674
	(0.102)	(1.175)	(1.196)
Siblings	−0.277	−4.422	−3.290
	(0.806)	(11.119)	(11.121)
Boarding	−6.638 *	−48.649	−38.335
	(3.705)	(41.426)	(41.538)
Left-behind Children	−0.466	33.027 ***	29.221 **
	(1.001)	(9.789)	(11.304)
Age of guardian	−0.061	0.329	0.411
	(1.347)	(0.463)	(0.439)
Education of guardian	−0.027	1.972	3.085 *
	(0.063)	(1.636)	(1.607)
Annual family income	0.033	−1.743	−1.432
	(0.122)	(1.215)	(1.425)
*N*	826	826	826
School FEs	Yes	Yes	Yes
Grade FEs	Yes	Yes	Yes
Class FEs	Yes	Yes	Yes

Note: Data from China Rural Children Health and Nutrition Survey (CRCHNS) in China on July 2019; *** *p* < 0.01, ** *p* < 0.05, * *p* < 0.10.
